# Enhancing landslide risk reduction strategies in Southeast Bangladesh

**DOI:** 10.4102/jamba.v15i1.1541

**Published:** 2023-12-22

**Authors:** Edris Alam, Md K. Islam

**Affiliations:** 1 Faculty of Resilience, Rabdan Academy, Abu Dhabi, United Arab Emirates; 2 Department of Civil and Environmental Engineering College of Engineering, King Faisal University, AlAhsa, Saudi Arabia

**Keywords:** landslide, hazard, risk reduction, preparedness, evacuation

## Abstract

Since 2000, landslides in southeast Bangladesh have resulted in over 700 deaths, most of which were in informal settlements. For instance, the 2007 landslides in informal settlements in Chittagong and the 2017 landslides in Rangamati took 127 and 150 lives, respectively. Although the government of Bangladesh claims to implement risk reduction, the number of deaths associated with landslides is increasing. Hence, this study investigated the ways to enhance landslide risk reduction interventions by addressing several scientific and technical issues related to disaster risk reduction. This study conducted focus group discussions, key informant interviews and expert interviews with key stakeholders in landslide risk management to collect qualitative data. Moreover, the historical accounts of settlements, media reporting, institutional action plans and policies were reviewed.

**Contribution:**

By integrating primary and secondary data, this study found that several political–economic aspects are major anthropogenic contributors to the recent increase in landslides in the region. The contributing factors included the following: policy and action plans to raise regional population; land management; illegal deforestation; plans to establish hydroelectricity in hilly areas; ownership of settlements; manipulation of water, gas and electricity supply to illegal settlements; commercial plantations; lack of risk governance; unplanned development activities; natural population rise; increased settlement along hill slopes. This study identified and discussed lessons learned from previous landslide disasters, the weakness of early warning systems and their dissemination and ways to improve evacuation, rescue, relief and risk reduction. Finally, this study formulated recommendations for the effective implementation of landslide risk reduction in southeast Bangladesh.

## Introduction and aims

The Government of Bangladesh (GoB) has been successful in reducing deaths from tropical cyclones; however, it faces another threat in the form of landslides, which put the lives of those residing in the southeast region of Bangladesh at considerable risk (Alam & Ray-Bennett 2021). Bangladesh has experienced an increase in the number of landslide-related deaths and injuries in the last two decades. Since 2000, landslides in southeast Bangladesh have resulted in over 700 deaths, most of which were in informal settlements. For instance, the 2007 landslide event in informal settlements in Chittagong and the landslides that occurred on 12 June 2017 in Rangamati took the lives of 127 and 150 people, respectively. The 2017 landslides occurred simultaneously in an extensive geographical location, including Rangamati, Bandarban, Chittagong and Cox’s Bazar (Sultana 2020). The governmental disaster risk management for this type of hazard was ineffective, and the effectiveness of improvements made following the 2007 landslide was questioned by the media, civil society and experts. The landslides in this region are attributed to natural and anthropogenic factors, including excessive and prolonged rainfall in short periods, unmanaged slope cutting, loose soil structure in hilly areas, deforestation in hilly areas and seismic activity (Ahmed 2021; Alam 2020; Chisty 2014). In particular, the unplanned hill cutting areas for formal and informal settlement development since the early 1990s have significantly contributed to landslide events.

In the 1960s, because of decreased habitation, landslides did not claim any lives in the southeast region and only caused damage in natural forest areas (Table 1). Landslide-related deaths were first recorded on 13 August 1999 when a landslide caused 27 deaths and damaged a 50 km road in Bandarban (Ahmed 2015). On 13 June 2017, landslides occurred at 145 locations along seven routes between Rangamati–Chittagong, Chittagong–Khagrachari and Khagrachari–Rangamati. In particular, at least 60 landslides on the Rangamati–Chittagong highway obstructed the supply chain from Chittagong; road transport was restored 8 days later. Moreover, road cracking was recorded in at least 22 locations on the Rangamati highway (Alam 2020; Sarwar & Muhibbullah 2022). Between 2012 and May 2017, landslides claimed the lives of 55 people, 53 of whom were non-tribal (Saha 2017).

**TABLE 1:** Catalogue of landslide events causing deaths and injuries in Bangladesh.

Year	Location	Deaths	Injuries
27 August 2023	Chittagong	2	2
05–10 August 2023	Chittagong	2	2
25 July 2018	Cox’s Bazar	5	1
03 July 2018	Bandarban	4	-
11 June 2018	Teknaf (kotukutuku)	1	500
11 June 2018	Rangamati: Naniarchar	11	-
30 December 2017	Rangunia	3	-
13 June 2017	Chittagong: Rangunia	21	-
13 June 2017	Chondonish, Chittagong	4	-
13 June 2017	Rangamati: Sadar	110	56
13 June 2017	Bandarban	6	-
2016	Chittagong area		100
19 July 2015	Moti Jorna and Nazir Pahar, Chittagong	6	Over 100
23 June 2015	DT Road Rail gate, Chittagong	2	-
03 February 2014	Panchlaish, Chittagong	1	2
03 February 2014	Panchlaish, Chittagong	1	2
2012	Lama, Laikhanchari, Bandarban, Chittagong, Moheshkhali	8	Over 100
26 June 2012	Lebubagan, Foyez Lake area, Chittagong	90	Over 150
2011	Batali Hill, Chittagong	17	Over 150
2010	Cox’s Bazar, Bandarban	53	Over 150
2009	Chittagong	3	8
18 August 2008	Moti Jorna and Lal Khan Bazar, Chittagong	11	15
10 September 2007	Nabinagar, Chittagong	2	-
11 June 2007	Moti Jorna, Lalkhan and Lebu Bagan	150	Over 100
3 August 2005	Nijam Road Housing Colony, Chittagong	2	12
19 June 2003	Patiya, Chittagong	4	-
24 June 2000	Chittagong University	13	20
13 August 1999	Lama Bandarban, Chittagong	27	-
1968	Kaptai, Chondroghona Road, Rangamati	No death	-

The GoB has been implementing landslide risk reduction strategies since 2000. Following the 2007 landslide event, the GoB formed an investigation committee to determine the causes and identify remedial processes. The investigation committee provided 36 recommendations comprising proactive and reactive measures for landslide disaster risk reduction. A technical committee provided another 30 measures to reduce the landslide risk in SE Bangladesh. The recommendations were to be implemented in the short, medium and long terms in cooperation with different organisations. The key recommendations were as follows: (1) forcing the eviction of illegal occupants in hilly areas and implementing afforestation activities in hilly areas; (2) stopping the lease of hilly areas by the GoB; (3) constructing guide walls; (4) constructing and improving drainage systems; (5) constructing boundary walls along fragile hills; (6) stopping the removal of sands from hills; (7) prohibiting the use of brick kilns within 10 km of hills; (8) prohibiting the construction of residential houses within 5 km of hills; (9) prohibiting the construction of residential houses along hill slopes and (10) taking legal actions against those responsible for hill cutting.

The implementation of these landslide risk reduction measures has been limited. The district administration has focused mainly on evicting occupants from high-risk areas during the rainy season and conducting evacuation based on high rainfall forecasts. Despite implementing disaster risk reduction measures, the number of deaths associated with landslides is increasing. Gaps may exist in the integration of landslide hazard science and practicalities from the perspectives of communities regarding landslide risk reduction, preparedness, evacuation planning and rescue and relief efforts in the region. To minimise deaths, injuries and damage associated with landslide hazards, efforts should be directed towards formulating practical solutions for the effective development and implementation of people-centred landslide risk reduction strategies.

Therefore, this study had the following objectives:

- Research objective 1: Identify the causes of the recent increases in landslide hazards in southeast Bangladesh.
- Research objective 2: Identify the challenges associated with landslide early warning, evacuation and rescue and relief operations in southeast Bangladesh.
- Research objective 3: Identify strategies to enhance landslide risk reduction in southeast Bangladesh.

In the following sections, the literature on landslide risk reduction strategies in Bangladesh is reviewed, and research gaps are identified. Subsequently, the research objectives and methodology of this study are presented, and the results are discussed accordingly. Finally, a series of recommendations for future research and the implementation of risk reduction strategies are summarised.

## Literature review and theoretical framework

The term ‘landslide’ refers to phenomena involving lateral and down slope movements of earth materials. Landslides may be classified according to their morphology, material and mechanism of initiation (Keefer 1984). They can also be defined as the movement of masses of rocks, debris or earth materials down a slope. Landslides are a type of ‘mass wasting’, which denotes any down slope movement of soil and rock under the direct influence of gravity. Slope movements come in five types – falls, topples, slides, spreads and flow (Cruden 2013). They are further subdivided according to the type of geological material (bedrock, debris or earth). Debris flows (commonly referred to as mudflows or mudslides) and rock falls are examples of common landslide types (Causes 2001).

Many studies have investigated the overlapping physical and social dimensions of landslide risk (Crozier 2005; Cruden 2013; Cui et al. 2021; Pal & Karnjana 2021). Landslide studies can be divided into three categories: (1) landslide hazard assessment, (2) social dimensions of landslide risk and (3) risk reduction and governance. Research on landslide risk reduction and governance in southeast Bangladesh has developed susceptibility maps and examined slope stability, types of slope failures and landslides associated with rainfall (Alam 2020; Chowdhury 2023; Hafsa, Chowdhury & Rahman 2022). Several studies from local, regional and international institutions have focused on hill cutting and associated landslide occurrences in Chittagong City (Ahmed 2015; Chisty 2014; Islam et al. 2014). Slope failure hazards in Chittagong City are attributed to heavy rainfall in loose soil structures in hill-cutting areas (Rabby, Hossain & Abedin 2022a). Heavy rainfall is one of the principal components of landslide hazard occurrence in this region (Alam, Sufi & Islam 2023; Islam et al. 2014; Khan et al. 2012). Landslide susceptibility maps for Chittagong City have been generated to implement informed risk reduction strategies (Ahmed & Dewan 2017; Ahmed et al. 2020). Alam et al. (2023) constructed an AI-based system to identify the correlation between landslide attributes, such as rainfall, mass area and elevation and landslide casualty and injury numbers.

Landslides cause deaths, injuries and damage to the infrastructure and housing of affected communities in the region. The burden of landslide effects is significant for low-income groups, who live in landslide-prone areas and often become victims (Rahman et al. 2022). The social dimensions of landslide risk, including land cover change, overpopulation, unplanned development activities and an increase in informal settlement along foothills, have been identified as causing the increased occurrence of landslides and associated deaths in southeast Bangladesh (Ahmed et al. 2023; Alam 2020; Rabby et al. 2022b). Community members reportedly perceive a low risk of landslides despite living in landslide-susceptible areas. For instance, the residents of Rangamati faced a landslide disaster for the first time on 12 June 2017. Despite early warnings of landslides, the locals were not concerned and did not relocate to safer places (Alam 2020; Rahman et al. 2022). Problems in early warning and evacuation systems are discussed in Sections ‘Landslide early warning’ and ‘Evacuation improvement’. The lack of landslide preparedness has been associated with several socioeconomic factors, including education, land and house ownership, ethnicity, and solvency of locals (Alam 2020).

Sultana and Tan (2021) and Alam and Ray-Bennett (2021) consulted key local administrators in southeast Bangladesh and concluded that although emergency response measures, including dissemination of early warnings and forced eviction and evacuation, are successful, improvements are required in early warning systems, sheltering facilities, mass community awareness and relief–rehabilitation processes. Similarly, Ahmed et al. (2023) consulted various key informants, including community leaders, disciplinary experts, civil administrators and elected representatives of local governments and identified short-, medium- and long-term measures. These measures included stopping illegal hill cutting and deforestation, regulating heavy vehicles, institutionalising early warning systems, conducting massive awareness campaigns, creating a master plan exclusive to the entire region and arriving at a political consensus for implementing the master plan effectively. This study contributes to landslide risk reduction by integrating the knowledge derived from consultations with local communities, conscientious citizens, elected local government representatives and civil administrators in the region.

Disaster risk may be defined as:

[*T*]he potential loss of life, injury, or destroyed or damaged assets which could occur to a system, society or a community in a specific period of time, determined probabilistically as a function of hazard, exposure, vulnerability and capacity. (UNISDR 2018:1)

Hazards can be triggered by natural sources or human activities and may result in a loss of lives, injuries, financial losses and overall environmental damage. Exposure refers to the characteristics of people, including their assets and livelihood and the built environment in hazardous locations (UNISDR 2018). Vulnerability is influenced by people’s perceptions, knowledge, and practices related to risk and disaster (Hilhorst & Bankoff 2004). To mitigate disaster risk and evaluate the frequency, intensity and duration of hazards, the varying characteristics of communities and economies that play a significant role in disaster vulnerability must be extensively explored (Ahmed 2021; O’Keefe, Westgate & Wisner 1976; Wisner et al. 2004). Specifically, the physical, social, economic, cultural, environmental and institutional dimensions of landslide vulnerability require comprehensive assessments (Crozier 2005).

## Materials and methods

### Research setting

**FIGURE 1:**
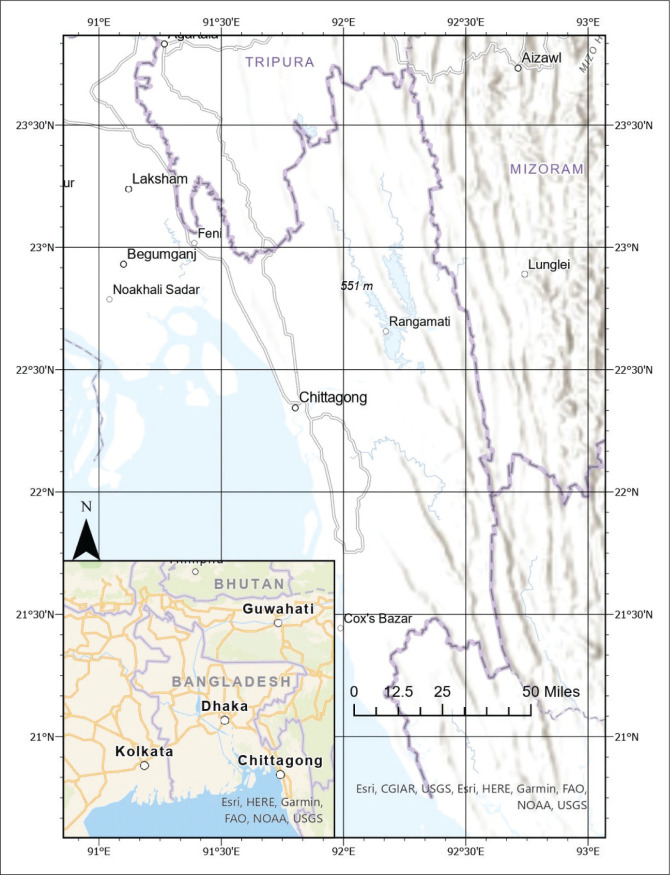
Location of southeast Bangladesh.

Referring to previous records of landslide events, this study selected southeast Bangladesh, including Khagrachari, Rangamati, Chittagong, Bandarban and Cox’s Bazar. These districts have a total area of approximately 19 888 km^2^ (BBS 2020). Except for the northeast Sylhet region, the southeast hilly districts are the major hilly areas in Bangladesh. They are bordered by Myanmar in the southeast, India in the north and northeast and the Bay of Bengal in the south and southwest (Figure 1). In this region, the high hills sub-region includes hill ranges with summits ranging from 300 m to 1200 m, whereas the low hills sub-region has summits ranging from less than 100 m to 300 m (Brammer 2012).

The southeast hilly region was formed by the convergence and collision of the Indian and Burmese plates and is currently situated on active plates. The layers of these hills are composed of anticline and syncline structures (Akhter 2017). These hilly areas can be divided into three landslide risk zones (i.e. high, medium and low) based on the compositional differences of the hills. Elongated hills elongated along the north–south, east–west and cross-sectional areas have many faults and cracks. Recent earthquake activities have further fragmented these faults and caused cracking. Every hill range has many mountains and valleys. Mountains are composed of hard rocks, whereas valleys comprise comparatively less hard rocks.

The hill ranges elongated in the north–south direction have slopes in the east–west direction. The stratified soils on the east–west side show anticline structures. The middle zone of the southeast hilly areas is generally high and comprises shell-type rocks. The eastern and western sides of the hill slopes merge into plains. These hilly areas are small and composed of sandy rocks. The areas in the middle of the two hilly areas are mainly composed of shells, silt and sand. This type of layering (i.e. shell, silt and sand) is highly susceptible to landslides. The region is vulnerable to climate change and increased precipitation over a short period (Islam et al. 2020).

A tropical monsoon climate prevails in the region from June to October, with a mean annual rainfall of nearly 2540 mm in the northwest and 2540 mm – 3810 mm in the southwest. Rainwater infiltrating the sand layer of these hilly areas is obstructed, reaching the shell layer and running downward. Consequently, the upper layer slips from the bottom, causing landslides. The recurrence interval of these types of landslides depends on the completion of subsequent weathering processes. Although the region has a record of historical and recent earthquakes (Alam & Dominey-Howes 2016), no known earthquake-induced landslides have been reported, and only rainfall-induced landslides are prominent in Bangladesh (Ahmed 2021).

The residents of high-landslide-risk areas in southeast Bangladesh can be divided into three distinct groups (Ahmed 2021). The first group includes Bengali communities who inhabited the plains but moved to areas vulnerable to landslides in the southeast region, including Chittagong, Cox’s Bazar and Rangamati. These communities were poor and landless while residing on the plains, and some of them moved to southeast Bangladesh after experiencing disasters. The second group are indigenous tribal people who are ancestral to the region and have been living in Bandarban, Khagrachari and Rangamati districts since ancient times. After the enforced settlement of Bengali communities led by the GoB in the early 1980s, land disputes and conflicts have arisen between the tribal people and Bengali population (Roy 2000). The tribal people live in houses raised on stilts and constructed of bamboo, wood and sun grass. The third group comprises the Rohingya refugees who fled from Myanmar to Bangladesh. As of 30 September 2023, the United Nations High Commission on Refugees (UNHCR) estimated the number of Rohingya refugees in Bangladesh as close to 965 467, including 934 719 living in Cox’s Bazar. The Rohingya refugees reside mainly in temporary overcrowded makeshift shelters made of bamboo frames, tarpaulin and plastic sheeting.

### Research approaches, data collection and analysis

This study aimed to explore landslide risk reduction strategies by consulting communities, experts and administrators within the domain of social science research. Qualitative and quantitative approaches are used in social research (Bryman 2008). Quantitative methods using experimental groups, statistical analysis and cause-and-effect relationships have been proved ineffective in exploring the underlying social interactions, complexity and diversity of human behaviour. In line with the research objectives and particular community settings, this study adopted a qualitative approach involving in-depth interviews and focus group discussions (FGDs). This approach helps gain in-depth knowledge from the subjects being studied through rapport building, discussion, interaction and mutual feedback processes (Bryman 2008).

This study consulted 59 key stakeholders in landslide risk management, including communities through FGDs and community leaders, experts and administrators through in-depth interviews between August and November 2018 (Table 2). Interviews aimed to identify the following: causes of landslides; political and ecological factors of landslide vulnerability; lessons learned from past disaster preparedness and response strategies; methods of improving landslide early warnings; measures to improve evacuation, rescue and relief operations; overall strategies for landslide risk reduction. Three separate FGDs were conducted with the residents of the Rangamati, Chittagong and Cox’s Bazar districts. The time, date and place of the FGDs were fixed prior to the consultations with the participants. The participants were informed of the purpose of the visits and discussions.

**TABLE 2:** Participant details.

Participant type	Interview type	Date conducted	Number of participants
Communities	FGD	August 2018	24
Community leaders/local conscientious residents, including local graduates	KII	September 2018	15
Elected representatives	KII	October 2018	3
District and sub-district administrators	KII	October 2018	12
NGO representatives	KII	October 2018	2
Subject experts	KII	November 2018	3

**Total**			**59**

FGD, focus group discussion; KII, key informant interview; NGO, non-governmental organisation.

Eight participants attended each discussion. Each FGD lasted approximately 1 h. At the beginning of each FGD, the event coordinator (the author of this study) described the objectives of the FGD and study goals. Subsequently, the coordinator disclosed the research ethics principles and obtained consent from the participants. Field research assistants took notes during the discussions. The scope of each FGD covered the three objectives of this research. Ethics approval was obtained from the University of Chittagong, Bangladesh, in compliance with the social research protocol for valuing participants’ rights and ensuring their informed consent.

The key informant interviews (KIIs) provided further insights into the knowledge gained from the FGDs on landslide risk and helped guide the improvement of the interventions for landslide risk reduction. Key informant interviews were conducted with 35 participants, including local conscientious residents and community leaders from tribal and non-tribal groups, elected representatives (e.g. city mayors, rural council members and local district and sub-district civil administrators), Fire Service and Civil Defence (FSCD) officials, police personnel, representatives of non-governmental organisations (NGOs) and three subject experts from environmental disciplines at the University of Chittagong, Bangladesh (Table 2). These key informants were consulted because of their understanding of landslide problems and involvement in decision making, GoB activities and execution of key preventive and response measures.

The study findings were shared with a wide body of academic, emergency management, disaster and health specialists at an international conference held in Canberra, Australia, on 23 October 2019. Moreover, the findings were shared with personal contacts in Chittagong and Dhaka. Following each disaster, Bangladeshi print newspapers extract expert comments, conduct roundtable discussions and continuously publish investigatory reports on causes, consequences and mitigation approaches for disaster risk reduction. This study extracted the findings from several news reports published immediately after the 2017 landslide event (Akhter 2017; Bal 2017; Karmakar 2017) and reviewed the policies, plans and institutional response mechanisms to validate the results of the FGDs and KPIs.

The data obtained from the KPIs and FGDs were checked and collated for analytical purposes. Subsequently, the responses were transcribed and translated into English. The results were mapped based on key contents aligned with this study’s objectives. The findings were interpreted by contrasting and comparing them with the existing literature. Finally, the results were used to identify effective landslide risk reduction strategies for Bangladesh.

### Ethical considerations

Ethical clearance to conduct this study was obtained from the University of Chittagong, Department of Geography and Environmental Studies (No. CUNI-13/0044).

## Results

### Perceived causes of increases in landslide hazards

Through field visits, observations and consultations with various stakeholders, this study identified 13 perceived causes, including 10 anthropogenic and three natural factors of landslide occurrence (Table 3). The anthropogenic factors included deforestation, hill cutting, changes in natural drainage, unplanned road construction, increases in settlement in adjacent areas, increases in settlement in slope areas because of migration, slash and burn cultivation and increases in population. For natural reasons, residents identified heavy rainfall as the main factor contributing to landslide occurrence, followed by earthquakes causing instability in soil, steep slope topography and thunderstorms.

**TABLE 3:** Causes of landslide occurrence.

Causes of landslides		Sources
**Anthropogenic causes**		
1.	Illegal human settlements in hill cutting areas and increases in human settlements in hill slopes	Expert interviews
2.	Lack of separate building code for Chittagong Hill Tracts (CHT)	Expert interviews
3.	Massive deforestation activities in hilly areas	Expert interviews
4.	Commercial plantation by cutting indigenous plants	KIIs
5.	Construction of the Kaptai hydropower plant by generating an artificial lake in early 1960s	Expert interviews and KIIs
6.	Construction of road transport and human settlement without considering the geological conditions of hilly areas	KIIs
7.	Lack of reinforced concrete walls along roads constructed by hill cutting	KIIs
8.	Increases in tribal and non-tribal populations in hill side and slope areas	KIIs
9.	*Jhum* (slash and burn) cultivation destroys top soil and causes an imbalance in soil cohesiveness	KIIs
10.	Setting up of deep tube wells in hilly areas	KIIs
**Natural causes**		
1.	Excessive rainfall in short periods	Expert interviews and FGDs
2.	Some key informants and residents considered thunderstorms the cause of landslides	KIIs and FGDs
3.	Small- to medium-sized earthquakes in Rangamati that may increase the faults in hilly areas	Expert interviews

The subject experts described that the recorded rainfall was 397 cm in Rangamati and 190 cm in Chittagong immediately before the 13 June 2017 landslide (BMD 2018). On 12 June 2007, a rainfall of 397 cm triggered landslides that caused 397 deaths in Chittagong. Heavy rainfall penetrates the soil, creating pressure on the subsoil; hilly soil becomes soft and loose and then slopes downward to the surface (Rahman 2017). The local residents, who live close to the landslide-prone areas in Rangamati, tend to panic when heavy rainfall events occur. From their experiences of recent landslides, the community members identified the occurrence of heavy rainfall for long periods as the cause of landslides in the area. For instance, the severe thunderstorm on the night of 12 June 2017 caused shaking in the hilly land areas. The participants explained that thunderstorms caused the ground to shake and led to landslides. Heavy rainfall coupled with thunderstorms caused loosening and fragmentation in hilly areas, leading to landslides. Severe thunderstorms, along with excessive rainfall, were recorded on the night of 12 June 2017.

**FIGURE 2:**
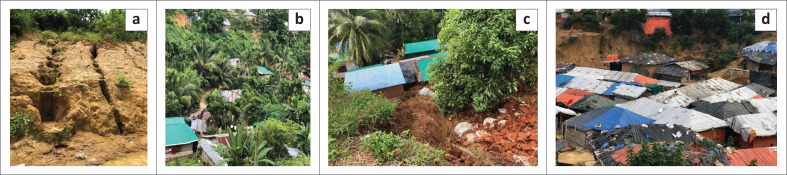
Fragile hills and settlement along hill slope in SE Bangladesh. (a) Loose hilly soil in Rangamati; (b) Settlement along fragile hills in Rangamati; (c) Landslides in Cox’s Bazar in 2017; (d) High-risk settlement at Kutu Palong Rohingya camp.

When hilly areas are covered by dense forests, rainwater quickly passes down the areas. Owing to increased deforestation over the last three decades, rainwater quickly infiltrates underground in hilly areas. The weathering process causes the fragmentation of rocks and increases the likelihood of landslide occurrence (Akhter 2017). Slope failure is likely if the slope of a hill is over 30° (Ahmed 2015). Owing to excessive hill cutting, many of the hill slopes in southeast Bangladesh are over 90° and are vulnerable to landslide occurrence. Additionally, the 615 mm rainfall recorded between 11 and 16 June 2017 was the highest in the last 40 years (BMD 2018). The soils in this area are soft and less cohesive, and excessive rainfall further aggravates this condition (Figure 2a). Excessive rainfall (e.g. 590 mm in 3 days) could infiltrate the hills through the faults and lead to landslides.

Houses, roads and infrastructure constructed along the anticline are highly susceptible to landslides and their associated risks. Road transport and human settlements in hilly areas were constructed without proper scientific studies, including an assessment of the geological conditions of hilly areas. Heavy vehicles that traverse these roads contribute to the increased risk of landslides. Human settlements are gradually increasing along these roads through the cutting of adjacent forests (Akhter 2017). From 2003 to 2015, at least 27.52% of natural forest was destroyed in the Chittagong Hill Tracts (CHT) region. This activity contributes to the slow fragmentation of hill slope areas, increasing landslide risk.

Hilly areas in southeast Bangladesh face deforestation because of the increase in settlement and commercial activities in the region. Interviews with experts and KIIs suggested that teak (*Segun*) tree cultivation in hilly areas, which involves cutting indigenous plants, inhibits the natural growth of other types of small trees and causes the soil structure to loosen. Commercial plantation (i.e. Gozari, teak, Euphorbiaceae [*Rubber*]) and fruit trees were cultivated via clearing of indigenous trees. The roots of these commercial trees are short and inhibit the natural growth of herbs and shrubs. Their cultivation creates loose and dry soil. Subsequent excessive rainfall then causes soil fragmentation.

Interviews with experts and KIIs addressed the construction of the Kaptai hydropower plant in 1960 and its expansion up to 725 km^2^. The original area of the lake was small. The extended lake areas submerged the valley area and caused changes in the soil structure (sand and silt) in the hilly areas. Rangamati used to be located in the plains; nonetheless, the construction of the embankment pushed people to the top of the hills. As this group of people was used to the plains and was unfamiliar with living in hilly areas, they caused damage to the hilly resources.

#### Multiple disasters increase landslide risk

The findings from the FGDs with the communities revealed that the participants moved to high-risk landslide areas after losing their homes because of disasters or conflicts for the second, third or even fourth time in their previous place of residence. The other reasons for relocating included low risk perception, low cost, limited alternative places to settle and advantages of the easy livelihood by relying on local forest, agricultural and lake-based sources. The KPIs with the local administrative officials in Cox’s Bazar revealed that the residents living adjacent to hills are mainly landless. Over time, those who failed to secure a place of residence in the plains shifted to high-risk areas along hill slopes. The FGDs in Chittagong showed that those displaced by disasters mainly live in high-risk areas. Mr Rahim, a participant of FGD at Moti Jorna in Chittagong, an area of high landslide risk, stated as follows:

‘I came to live here when I lost my father’s house in Sandwip Island due to erosion near the Bay of Bengal. I do not have money to move from here to another location without direct support from the GoB or NGO providing me a safe land and constructed house.’ (Rahim [39], a rickshaw puller, a male participant of FGD)

The residents in Rangamati considered their place of residence to be relatively safe, as they did not experience frequent tropical cyclones, erosion or flooding like inhabitants in plains. However, the 2017 landslides proved their mistake. The settlement process by multiple-disaster experienced residents in high landslide risk hilly slopes can be understood from an extensive interviews with Mohammad Rafiq, a male auto-rickshaw puller in Rangamati.

‘Mohammad Rafiq (36) lost his house in Companiganj along the Bay of Bengal coast because of coastal erosion 10 years prior and then moved to Chittagong to find a job. He drives a battery-run auto rickshaw. Rafiq’s wife works in a garment factory in the Bayzid Bostami area. His son Yunus studies in a madrasa (Muslim school) located in Jungle Salimpur. Rafiq has not been able to secure a safe place in Chittagong even after living there for six years. He managed to construct a house cutting a hill slope in Jungle Salimpur two years back. Around 20 to 30 houses were constructed adjacent to his house in that area. Specifically, Rafiq’s house was constructed over the top slope of the hills. To construct a house in such a high-risk area, Rafiq had to pay 1 lac (US$ 1,000) to the political leaders. Currently, Rafiq is being asked by GoB officials to vacate his house. Although the house is indeed located in a high-risk area, he does not want to move because they had used all their earnings and savings to occupy the land and construct their house.’

#### Landslide early warning

Following the 2017 landslide, the GoB has been routinely disseminating early warning messages to landslide-prone areas. Although local district officials have attempted to disseminate warning signals to communities through loudspeakers, community members complain that the announcer communicates hurriedly and then leaves the area before they can understand the message. Some residents reported not receiving any warning at all. Owing to high wind speeds and heavy rainfall, early warnings do not always reach all residents. Moreover, local government organisations send warnings at the last minute, whereas they should do so as early as possible based on rainfall prediction. Community members emphasised the need to improve the dissemination of early warnings of landslides in each locality through loudspeakers. For instance, officials should deliver a clear message and take adequate pauses when passing each locality to help residents understand the warnings and recognise possible dangers (Table 4). During disasters, the locals living in remote areas do not receive landslide warnings. District and sub-district leaders should place permanent signs of possible landslide events in high-risk areas. The community members explained that they prefer to receive warnings about landslides through mobile text messages, TV broadcast and social media (e.g. Facebook).

**TABLE 4:** Suggestions to improve effective early warning systems.

Warning system improvement		Sources
1.	Early warnings through loudspeakers should reach every locality, including remote areas	FGDs
2.	Early warnings should be disseminated via television broadcast	FGDs
3.	Early warnings via loudspeakers should be provided by local police	FGDs
4.	Awareness during regular periods should be promoted	KPIs
5.	Shelters should be provided in safe zones	FGDs
6.	Early warnings should be disseminated via mobile text messages and social media	FGDs
7.	Warnings should be disseminated in places of worship, including mosques	KPIs

FGD, focus group discussion; KPI, Key informant interview.

#### Evacuation improvement

The KPIs with administrative staff revealed that communities tend to refuse to evacuate prior to landslide emergencies, with many of them stating, ‘If we die, we will die at our own house’. This attitude is the main obstacle to executing a successful evacuation prior to landslide occurrence. The FGDs with communities revealed that residents had negative experiences in shelters, such as public schools and municipal halls. Shelters usually exceed capacity; sleeping areas and toilet facilities are limited, and the shelters cannot accommodate thousands of evacuees. One participant stated, ‘I have a young daughter and do not want her to evacuate to a shelter with no private room where she may experience sexual harassment’. Hence, leaders should seek each community’s acceptance of shelters before attempting to evacuate them (Table 5). Community members would likely agree to evacuate and move to shelters if they were guaranteed the provision of food, medicine, separate rooms for women and transport arrangements for evacuation. The engagement of community leaders and improved awareness could facilitate evacuation efforts. Some of the residents reported experiencing theft at their homes when they went to the shelters. Many tribal communities suggested the provision of separate places in shelters or separate shelters for tribal and non-tribal communities. A tribal participant stated, ‘I do not want to go to a shelter centre because I do not panic about landslides. If I go, my house may get robbed’.

**TABLE 5:** Suggestions to improve evacuation.

Generate confidence among local residents about various options for shelter Ensure adequate food supply in shelters Improve road transport for evacuees Ensure sufficient space, medicine supply and toilet facilities in shelters Construct disaster shelters in agreed location with communities Effectively cooperate with local communities in conducting evacuations Generate awareness

#### Improving post-disaster rescue, relief and rehabilitation

This study identified 17 measures to improve landslide-related rescue, relief and rehabilitation in the region (Table 6). The FGDs with the local residents and the KPIs with the community leaders revealed that the food supply is insufficient and that people have reported awkward experiences in the shelters. The community members also explained that the governmental and NGO officials involved in relief and rehabilitation in the area lacked professionalism and honesty. They claimed that they did not receive the relief items allocated to them. Moreover, they highlighted the limited role of governmental organisations in remote areas. Participants described how the local administration attempted to force them to leave their residences without prior consultation and provision of alternative places to live and secure new livelihoods. Furthermore, the results of the KPIs suggest that strengthening health departments may help reduce deaths during disasters.

**TABLE 6:** Methods of improving post-disaster rescue, relief and rehabilitation.

Ensure prompt rescue efforts Ensure the availability of equipment and logistics for rescue at the local level Train local people about the basics of rescue and engage them in emergency rescue operations Promote collaborative efforts among locals, police and defence personnel Strengthen fire services and effectively engage them during emergencies Provide immediate food and water to affected people Treat all victims equally during relief distribution Provide financial aid to affected people Provide medicine to affected people Generate awareness through training in disaster risk reduction Relocate affected people to safe areas Construct houses for victims Improve road transport and communication Effectively monitor rescue, relief and rehabilitation activities by the central government Construct retention walls along fragile hilly areas Implement training on the construction of disaster-resilient houses Generate employment opportunities

The communities expect that governmental organisations would make effective rescue and relief efforts during the post-disaster period (Table 6). They stated that they could do little before the arrival of rescue and relief teams from the outside. The 2017 landslide completely destroyed the road transport system in Rangamati. It also cut off the supply of electricity and water in the city. The FSCD in Rangamati lacked the workforce and technology to handle such a disaster. The locals described how the personnel of the FSCD failed to rescue the residents trapped underneath the soil during the landslide event. Therefore, rescue efforts should be improved to protect people’s lives.

As rescue teams require a long time to reach disaster areas, local residents should be trained on basic rescue efforts and encourage their engagement. During the rescue efforts following the 2017 landslides in Rangamati, the locals directed the rescue personnel to the specific locations where people could be trapped. Local residents were the first responders who rescued their neighbours following the landslide event. In the aftermath of the 12 May 2008 Wenchuan earthquake (magnitude [Mw], 8; intensity [Ms], 11), 87 000 people were rescued. Of these, 70 000 were rescued by self-help and mutual aid groups, whereas the remaining 7000 people were rescued by professional rescue teams (Shen et al. 2015). The number of people rescued by self-help and mutual aid groups was 9.4 times higher than that rescued by professional teams. Only 30% of the local people were not involved in the Wenchuan earthquake rescue. Following the earthquake, the central government focused on public awareness of self-help and mutual aid for disaster risk management. During the 2013 Yushu earthquake, 96% of the locals were involved in the rescue efforts (Shen et al. 2015). Search and rescue teams involving locals in landslide risk areas in Bangladesh have not been developed to date.

## Discussion

This study identified the causes of the recent increases in landslide and challenges associated with landslide early warning, evacuation and rescue and relief operations in southeast Bangladesh. Moreover, strategies to reduce landslide risk in southeast Bangladesh were identified. The identified factors included the following: policy and action plans to raise the population in CHT; unplanned road transport and settlement development; plans to establish hydroelectricity systems in hilly areas; hill cutting; deforestation; changes in natural drainage; provision of land ownership to individuals; manipulated water, gas and electricity supply to illegal settlements; ownership of settlements by politicians; commercial plantations; poor governance in infrastructure development; corruption in disaster risk reduction activities. Increases in anthropogenic factors have contributed to the occurrence of landslide hazards in Bangladesh (Ahmed et al. 2022, 2023), India (Jones et al. 2021) and China (Zhang et al. 2023). In particular, Ahmed (2021) and Ahmed et al. (2023) observed an increase in Rohingya refugee settlements, which are highly exposed to landslide risk across southeast Bangladesh. Additionally, sharp increases in tribal and non-tribal populations have been observed in hill side and slope areas, which have exacerbated exposure to landslide risk in the region. According to the 2011 census, 51% of the population were tribal individuals, compared to 9% in 1960 and 41% in 1980 (BBS 2020).

A review of past landslide relief efforts also provides important lessons for future improvement. In the aftermath of the landslides in 2017, governmental organisations and NGOs played an important role in conducting prompt rescue and relief operations in Rangamati. The local district and sub-district officials were active in the post-disaster period under the direct guidance of the different ministries and the Prime Minister Office. Over 200 NGOs and community-based organisations (CBOs) provided relief either directly or through government-run relief operation centres. Thousands of individuals sent help, including financial assistance and clothing, to the affected people through various governmental organisations, NGOs and CBOs. The GoB has attempted to implement the same process immediately after disasters occur. However, organisations tend to disregard landslide disaster preparations over time. Effective post-disaster monitoring strategies are clearly lacking. This study identified the lack of effective implementation of recommendations for landslide risk management. For instance, limited preventive and preparedness activities were observed in the Rangamati Hill District. These findings were in line with those of Ahmed et al. (2023) and Alam and Ray-Bennett (2021), who identified anthropogenic aggravators and failed disaster risk governance relating to landslides, respectively.

The continued development of settlements and population growth in high-landslide-risk areas necessitate limiting the exposure of communities and their assets and livelihoods to landslide disasters. Reducing exposure to landslide hazards requires relocating people from high-risk areas to avoid future deaths, injuries and property losses because of disasters. Relocation sites should be developed with full consideration of the opinions of local communities and acceptable livelihood options (Sultana & Tan 2021). Although many residents had observed the extensive damage caused by previous landslides adjacent to their areas, they consider their places of residence relatively safe. Disaster preparation has been found to be dependent on the duration of residence, ownership of houses, ethnicity, gender and economic status of residents (Ahmed 2021; Alam 2020). The GoB actively initiates disaster risk reduction activities immediately after the occurrence of destructive events. Nevertheless, such efforts are mostly short-term, whereas effective long-term disaster risk reduction approaches are clearly lacking. Thus, efforts must be undertaken to consider vulnerability factors, particularly local residents’ perceptions of landslide disasters and risks and landslide disaster risk reduction strategies (Alam 2020; Sarwar & Muhibbullah 2022). Furthermore, monitoring and evaluation activities must be improved to ensure an effective landslide disaster risk management. This study suggests formulating and implementing robust landslide risk reduction strategies, including continuous improvements in early warning systems, evacuation, response, relief and rehabilitation following landslide occurrence.

Based on a comprehensive analysis of documentary sources and data gathered through field research, this study prepared a comprehensive list of preventative, preparedness, response, rescue and rehabilitation plans to improve landslide risk management in Bangladesh (Table 7). A bottom-up planning process is required to reduce landslide risk effectively. The study participants reported that government officials often visit highly affected communities and impose risk reduction implementation strategies without conducting participatory consultation processes. A total-community approach that involves all stakeholders in individual landslide-prone areas is undeniably important.

**TABLE 7:** Recommendations to enhance landslide risk management.

Recommendations to enhance landslide risk management		Sources
**Hazard and risk assessment**		
1.	Identify existing landslide risk areas and implement risk reduction measures	Expert interviews
2.	Evaluate community vulnerability to landslide disasters	Expert interviews
**Landslide prevention**		
3.	Organise community mobilisation meetings in regular periods and increase public awareness to stop hill cutting and deforestation and avoid living in high-risk areas	KPIs
4.	Install retention walls and wire meshes in high landslide risk areas	Akhter (2017)
5.	Strengthen the side walls of existing fragile roads and hills by constructing reinforced concrete walls	KPIs
5.	Construct shelters in safe locations to increase local communities’ confidence in going to these shelters	Community survey and KPIs
6.	Continuously monitor the hilly areas to stop hill cutting and prohibit the development of new settlements along hill slopes	Local communities and community leaders
7.	Transform the ad-hoc and reactive activities of the GoB into proactive and comprehensive activities to implement risk reduction	KPIs
8.	Promote afforestation to reduce erosion and increase the compactness of soil in hilly areas	Expert interviews
9.	Improve road transport systems for safe evacuation operations	Local communities
10.	Improve drainage systems along hill slopes and roads	Akhter (2017)
11.	Strengthen participatory hill management and monitoring to stop the construction of new houses adjacent to hills	Expert interviews
12.	Construct houses in the CHT region in accordance with the Bangladesh National Building Code, which is mainly for the plains (Saha 2017)	Saha (2017) and expert interviews
13.	Ensure that only bamboo houses are constructed in hilly areas, as some varieties of trees (e.g. coconut, *Acacia auriculiformis* [*Akashmoni*], *Albizia lebbeck* [*koroi*], jackfruit, *Areca catechu* [*supari*], fulkumari and bamboo) have deep roots that can strengthen soil cohesiveness	Local communities
14.	Ban new settlements in hilly areas	Tribal residents
15.	Declare hilly areas as reserve areas	Expert interviews
16.	Install permanent signs of possible landslide hazards in high-risk areas	Local communities
17.	During road construction, ensure that water does not penetrate the ground and that appropriate drainage is provided to reduce the occurrence of landslide along roads	Local communities
18.	Undertake and execute comprehensively studied long-term development plans by considering the geological characteristics of hilly areas (e.g. morphology, slope, angle and characteristics of soil)	Expert interviews
19.	Local governmental organisations (e.g. DC, UNO) must take actions routinely to disconnect illegal water, gas and electricity supply to communities living in high-risk areas	Bal (2017); Karmakar (2017)
20.	The GoB should offer alternatives to Jhum cultivation	Expert interviews
21.	Encourage the active involvement of local communities in disaster risk reduction	Local communities
**Landslide preparedness and early warning implementation**		
22.	Implement a bottom-up planning process to ensure the coverage of all strata of local communities for disaster preparation and response	Local communities
23.	Educate people about landslides using leaflets and videos	KPIs
24.	Ensure the availability of assistance for people with disabilities	Local communities
25.	Ensure preparedness in regular periods, including undertaking evacuation drills and exercises	Expert interviews
26.	Form local search and rescue committees and train locals in first aid	This study
27.	Employ mobile text messaging for early warnings	KPIs
**Coordination, collaboration and implementation**		
28.	Enhance the coordination between civil administration, police, railway department, local government and engineering departments and power development board	This study
29.	Improve logistics and workforce to ensure the quick restoration of road transport systems and supply chain	This study
30.	Deploy local volunteer teams for evacuation and rescue and relief operations	Local communities
31.	Implement the recommendations derived from the experiences during the 2007 landslide event and share them with different concerned ministries (e.g. Department of Public Works, LGED)	This study
**Rescue-relief**		
32.	Deploy helicopters during post-disaster rescue operations for prompt response	Local communities
33.	Strengthen FSCD by deploying more workforce and increasing technical capacity for search and rescue operation	Local communities and KPIs
34.	Ensure alternative livelihood during regular periods prior to implementing relocation operations during high-risk periods	Local communities
**Relocation and rehabilitation**		
34.	Utilise existing social networks for warning dissemination, evacuation and post-disaster relief and rehabilitation	Key informant interviews
35.	Develop possible separate relocation areas with livelihood options for tribal and Bengali communities living in high-risk areas in consultation with them	Local and Bengali communities
36.	Ensure resilient livelihoods in relocated areas	KPIs
**Monitoring and evaluation**		
37.	Ensure that governmental disaster risk reduction activities are continuously conducted in all risk areas, as such activities tend to accelerate only after a major disaster (e.g. after the landslides of 2007 and 2017) and slow down in other periods, particularly in low- and moderate-risk areas.	This study
38.	Develop a monitoring plan and evaluate the implementation of recommendations	This study

CHT, Chittagong Hill Tracts; GoB, Government of Bangladesh; FSCD, Fire Service and Civil Defence; DC, Deputy Commissioner; UNO, Upazilla Nirbahi Officer; LGED, Local Government Engineering Department.

This study had some limitations. The findings presented were based on interviews and FGDs with stakeholders from local communities and administrators in southeast Bangladesh. Although these findings were fundamental in formulating landslide risk management strategies, they must be cross-checked for effectiveness and acceptance at the community level before attempting implementation. Therefore, future research should include whole community interactions, particularly face-to-face iterations between local communities, leaderships and government representatives, as well as regional civil administrators.

## Conclusions

This study identified the causes of the recent increases in landslide hazards, risk management challenges and methods of enhancing landslide risk reduction in southeast Bangladesh. By integrating the findings of a review of secondary and primary data sources, this study demonstrated that community-based factors, governance aspects and institutional failures have contributed to landslide risk and associated deaths in Bangladesh. The findings indicated that political–economic aspects of land, environmental and disaster management play an active role in landslide disaster risk reduction in southeast Bangladesh. The results revealed that governmental disaster risk reduction strategies are active prior to monsoon periods and after disaster periods. Hence, this study suggests implementing disaster preparation activities year-round to develop landslide-resilient communities. Disaster preparedness can be significantly improved through the effective dissemination of warning signals to local communities through microphones and loudspeakers, mobile text messages and social media (e.g. Facebook and Twitter). To execute the evacuation of local communities during high-risk periods, acceptable and appropriate shelters should be constructed in consultation with local communities. This study summarised lessons learned from previous landslide disasters, weaknesses of early warning systems and their dissemination and methods of improving evacuation, rescue, relief and risk reduction. Finally, this study formulated recommendations to improve early warning systems and their dissemination and provided key strategies to improve evacuation, rescue, relief and effective implementation of landslide risk reduction in southeast Bangladesh.
